# Antimicrobial potential of lactic acid bacteria from domestic chickens *(Gallus domesticus)* from south Celebes, Indonesia, in different growth phases: *in vitro* experiments supported by computational docking

**Published:** 2020-02

**Authors:** Dirayah Rauf Husain, Syahrul Gunawan, Sulfahri Sulfahri

**Affiliations:** Department of Biology, Faculty of Mathematics and Natural Sciences, Hasanuddin University, Makassar, Indonesia

**Keywords:** Lactobacilli, Probiotic bacteria, Surfactin

## Abstract

**Background and Objectives:**

Pathogenic bacterial infection is one of the factors that can cause extensive losses in poultry farming. Pathogenic bacteria that infect domestic chickens *(Gallus domesticus)* include *Escherichia coli*. This study has investigated antimicrobial compounds from probiotic bacteria isolated from the digestive tract of domestic chickens originating from Takalar Regency, South Sulawesi, Indonesia.

**Materials and Methods:**

Lactic acid bacteria were grown on de Man–Ragosa–Sharpe agar medium for 24 hours. The bacterial isolate with the best inhibitory power was identified as *Bacillus subtilis (B. subtilis)*, based on 16S RNA sequences. Antimicrobial activity of the selected lactic acid bacteria was tested on the pathogenic bacteria, *E. coli* and *Staphylococcus aureus*. Using well diffusion method. In this study, in silico study was conducted to examine the structure and binding affinity of lactic acid bacteria against *E. coli* and *S. aureus*. Molecular docking experiments were performed using the PyRx 0.8 software.

**Results:**

This study showed that the bacteria were *B. subtilis* strain PATA-5. The response of inhibition of antimicrobial compounds produced by *B. subtilis* strain PATA-5 maximum in the stationary phase. The bactericidal properties of *B. subtilis* strain PATA-5 were categorized as strong against Gram-negative *E. coli*, i.e., 30.5 mm, when compared to Gram-positive *S. aureus*, i.e., 17.5 mm.

**Conclusion:**

*B. subtilis* strain PATA-5 is capable to produce natural antibiotic cyclic lipopeptides, namely surfactin.

## INTRODUCTION

Pathogenic bacterial infection is one of the factors that can cause extensive losses in poultry farming. Pathogenic bacteria that infect domestic chickens *(Gallus domesticus)* include *Escherichia coli* (1). *E. coli* can cause growth disruption, decreases production and reduces the quality of meat and eggs, which often results in disease and high economic losses (2). Other bacteria such as *Staphylococcus aureus* are also a cause of acute infection in livestock chickens (3). Infections by *S. aureus* in chicken feet can cause bumble foot (4) and can also cause enterotoxins to accumulate to harmful levels in chicken meat (5).

The use of antibiotics is one effort to overcome and prevent pathogenic bacterial infections in broiler chicken farms. However, antibiotic use can cause deficiency and disruption to the natural defense mechanism of the gastrointestinal microflora, and resistance to pathogenic bacteria (6). Human consumers of broiler chickens can also be exposed to and suffer deleterious effects of antibiotic use through residues left on meat and egg products (7). However reducing the use of antibiotics in livestock can only be achieved if alternative antimicrobials are available.

One alternative antibiotic product for livestock are feed additives known as probiotics (1). Probiotics are beneficial microbes that have benefits in maintaining digestive microbial LAB and have a positive influence on the physiology and health of the host (8). The effects that probiotics can provide include modulating the host's immune systems through colonization and adhesion of the intestinal mucosa (4, 9, 10), increasing the efficiency of the digestive process and absorption of food nutrients by influencing villus ileum height (11).

One common type of probiotic bacteria are lactic acid bacteria; some of them bacteria have the potential as probiotics that are beneficial for the growth of broilers (12). The source of potential lactic acid bacteria can come from outdoor-raised domestic chickens from Indonesia because their habitat in the wild allows high levels of biodiversity of bacteria in their digestive tract. This study aims to investigate the potential for developing probiotics from lactic acid bacteria derived from the gastrointestinal tract of domestic chickens from Takalar, South Sulawesi, Indonesia in inhibiting the pathogenic bacteria *E. coli* and *S. aureus.*

## MATERIALS AND METHODS

**Isolation of probiotic bacteria.** A sampling of domestic poultry *(G. domesticus)* was conducted at Takalar, South Sulawesi, Indonesia. The inner walls of the chicken intestine were scraped and then inserted into a sterile NaCl solution and serially diluted into separate samples. De Mann–Rogosa–Sharpe agar (MRSA) medium was inoculated with 1 mL of the dilutions and 1% CaCO_3_ was added, then the medium was incubated for 24–48 h at 37°C.

**Purification, morphology and making stock isolates of probiotic bacteria.** Purification of bacteria was carried out by selecting of a single colony that was surrounded by a clear zone in the MRSA medium and incubating it at 37°C for 48 h. The morphology of each colony formed after purification was then observed. Each of the different colonies formed after purification was then inoculated on a slant MRSA medium for further testing.

**Resistance to gastric acidity, bile salts and pathogenic bacteria inhibitory test.** Resistance to acidity was tested using de Mann–Rogosa–Sharpe broth (MRSB) medium supplemented with 0.1 N HCl to obtain pH 2.5–3.0 (i.e., the pH of the chicken stomach). Resistance to bile salts was tested using MRSB medium supplemented with synthetic bile salts (ox bile) at concentrations of 1% and 5%. A total of 1 ooze from each bacterial isolate was taken from the stock culture and used to inoculate the MRSB-bile salts medium. The inoculated media were then incubated for 2–3 h at 7°C. The number of bacterial colonies growing before and after incubation was measured. The pathogenic bacteria inhibitory test was tested on *E. coli* and *S. aureus* using a well-diffusion method.

**Identification of lactic acid bacteria.** Molecular identification was utilized to identify the PATA-5 strain. 16S rDNA of the selected isolates were amplified by PCR using primers 27F (5′-AGAGTTTGATCCTGGCTCAG-3′) and 1492R (5′-TACGGYTACCTTGTTACGACTT-3′). All obtained sequences were screened via the BLAST program (https://blast.ncbi.nlm.nih.gov/Blast.cgi). The sequencing results for PATA-5 had a 100% query cover and 99% similarity with *Bacillus subtilis*.

**Preparation of lactic acid bacteria and pathogenic bacteria.** *B. subtilis* strain PATA-5 was cultured in MRSB media and incubated for 24 hours at room temperature at 120 rpm using a rotary shaker. Meanwhile, *E. coli* and *S. aureus* were etched on NA oblique media and incubated for 24–48 hours at 37 °C.

**Measurement of lactic acid bacterial growth curve.** A growth curve for *B. subtilis* PATA-5 was made by inoculating a 24-hour-old culture in MRSB media, then incubated for 24 hours at room temperature at 120 rpm using a rotary shaker. The measurement of bacterial growth was carried out using a spectrophotometer with a wavelength γ = 580 nm at three-hour intervals.

**Inhibitory power test for pathogenic bacteria.** Inhibitory tests of *B. subtilis* PATA-5 were carried out in the exponential- and stationary phase based on the growth curve. Inhibitory properties of PATA-5 were also compared with tetracycline (30 ppm) as a positive control. The inhibitory power test was carried out by diffusion method by using a 6-mm blank disk. A total of 1 ml of *E. coli* and *S. aureus* isolates were inoculated on NA medium with pouring method and allowed to solidify. A sterile disk blank allowed to soak in either *B. subtilis* strain PATA-5 culture media or a tetracycline solution for 10 minutes. The blank disk was placed on the surface of the NA medium that had solidified, then incubated for 24 hours at 37 °C. The diameter of the clear zone (mm) that was formed was measured using a caliper.

**In silico study.** Structures of potential chemical candidates of *B. subtilis* strain PATA-5 antibiotics were collected from published literature. Chemical 3D structures and the SMILES strings were obtained from the PubChem compound database (https://pubchem.ncbi.nlm.nih.gov/). The protein potential target candidates for docking were prepared using three databases, namely: Pharmmapper (http://www.lilab-ecust.cn/pharmmapper/), SuperPred (http://prediction.charite.de), and Swiss Target Prediction (www.swisstargetprediction.ch) and validated using Uniprot (https://www.uniprot.org). Molecular docking experiments were performed using the PyRx 0.8 software. The reverse docking process was carried out using the Vina Wizard feature integrated into PyRx 0.8. Compound activators were positive control in the docking process and visualized and analyzed using PyMol. The PA (probable to be active) value was predicted by the Way2Drug PASS server.

## RESULTS

**The morphology of isolated probiotic bacteria.** There were 12 isolates which were observed on medium. Gram staining showed that these 12 isolates had various morphology as listed in Table 1. Based on the resistance to gastric acidity test, resistance to bile salts analysis, and pathogenic bacteria inhibitory test on these 12 probiotic bacteria indicated that the best lactic acid bacteria isolated was PATA-5 (data not shown). Furthermore, PATA-5 was selected for further analysis.

**Table 1.** The isolated probiotic bacteria from domestic chicken *(Gallus domesticus)*

**Isolate**	**Shape**	**Gram type**
PATA-1	Rod	Gram-negative
PATA-2	Rod	Gram-negative
PATA-3	Coccus	Gram-negative
PATA-4	Coccus	Gram-negative
PATA-5	Rod	Gram-negative
PATA-6	Rod	Gram-negative
PATA-7	Rod	Gram-negative
PATA-8	Coccus	Gram-negative
PATA-9	Coccus	Gram-positive
PATA-10	Rod	Gram-negative
PATA-11	Rod	Gram-negative
PATA-12	Rod	Gram-negative

**Fig. 1.**
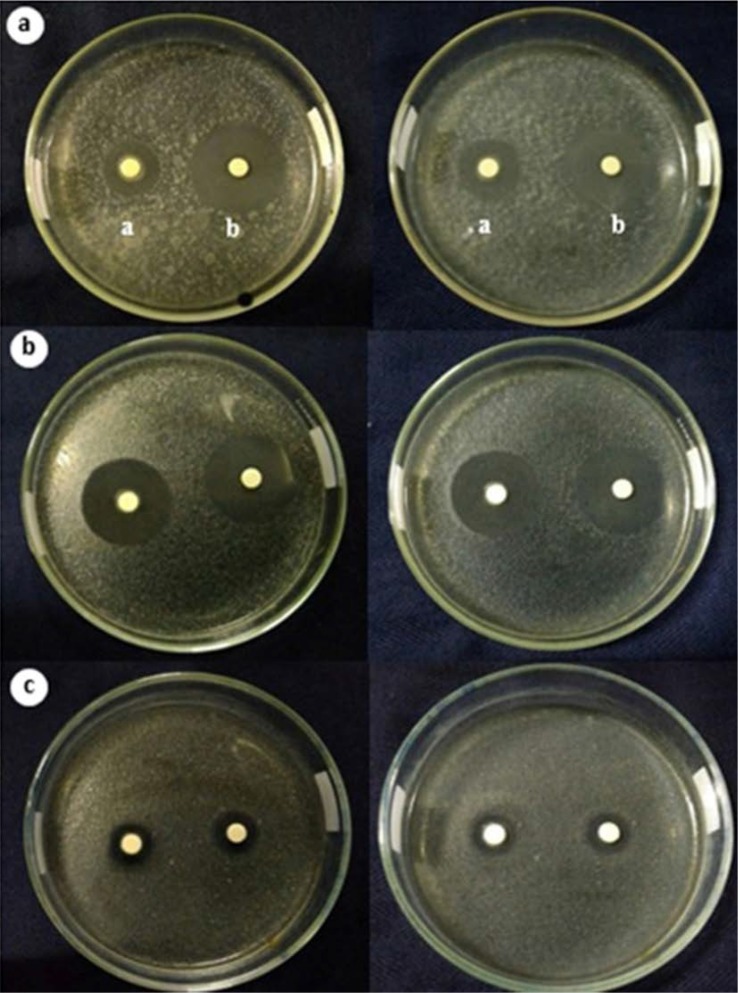
*B. subtilis* strain PATA-5 culture media inhibition of *E. coli* and *S. aureus* in the exponential (24 hours growth) and stationary phases (48 hours) of the inhibitor bacteria. (a) Left—*B. subtilis* strain PATA-5 in exponential phase, right—stationary phase; (b) Left—*B. subtilis* strain PATA-5 in exponential phase, right— stationary phase; (c) Left: 30 ppm tetracycline positive control at 24 h incubation time, right: 48 h incubation time.

**Inhibitory power of *B. subtilis* strain PATA-5 against pathogenic bacteria.** The ability of the PATA-5 strain and tetracycline in inhibiting the growth of these bacteria is presented in Fig. 1.

Fig. 1. shows that *B. subtilis* strain PATA-5 and tetracycline have bactericidal properties against *E. coli* and *S. aureus* at 24 hours and 48 hours incubation, demonstrated by the firm periphery of the inhibition zone. The comparison of the size of the inhibitory zone produced by *B. subtilis* strain PATA-5 and tetracycline against *E. coli* and *S. aureus* is presented in Table 2. The strength of the antibacterial effects were classified into four categories based on the diameter of the clear zone formed, namely, weak (<4 mm), medium (4–8 mm), strong (8–12 mm), and very strong (>12 mm) (Oldak et al. 2017). The diameters of the clear zones presented in Table 2 show that *B. subtilis* strain PATA-5 under exponential phase (7 hours incubation) and stationary phase (21 hours incubation) had a strong response to *S. aureus* and are very strong against *E. coli*, while the tetracycline antibacterial power criteria are categorized as very strong in inhibiting both *E. coli* and *S. aureus*.

**Table 2.** Comparison of inhibitory zone of *Bacillus subtilis* strain PATA-5 and tetracycline against *E. coli* and *S. aureus*

**Bacteria**	**Incubation Time**	**Average diameter of inhibitory zone (mm)**			

		***Escherichia coli***		***Staphylococcus aureus***	

		**24 h**	**48 h**	**24 h**	**48 h**
*B. subtilis*	7 h	21.5	21	11	10.5
tetracycline	7 h	32.5	32	16	16
*B. subtilis*	21 h	28.5	28	10.5	10.5
tetracycline	21 h	30.5	30.5	17.5	17.5

The antimicrobial activity of *B. subtilis* strain PATA-5 and tetracycline were found to be higher against the gram-negative bacteria *E. coli* than the gram-positive bacteria *S. aureus.* The results obtained in this study indicate that *B. subtilis* strain PATA-5 isolated from the digestive tract of domestic chickens from Takalar showed an inhibitory spectrum that was not much different from tetracycline antibiotics in inhibiting *E. coli* and *S. aureus*. This potential can make *B. subtilis* strain PATA-5 a natural preservatives as well as a source of alternative antibiotics that are safe for consumption for livestock and humans, replacing synthetic tetracycline antibiotics.

**Fig. 2.**
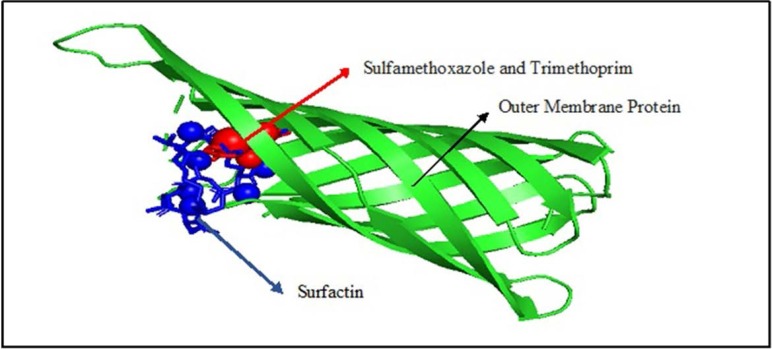
Binding site of surfactin (blue), sulfamethoxazole and trimethoprim (red) with outer membrane protein (green).

**In silico study.** Docking results predicted a binding site of surfactin with outer membrane protein, as shown in Fig. 2.

Molecular docking was modeled by blind docking. The more negative the binding affinity value, the easier it is for ligands and receptors to bind. In this case, surfactin has a score of −6.2, which is the same as sulfamethoxazole and trimethoprim; as such, surfactin is predicted to have potential as an antibacterial. Surfactin potential is reviewed based on the PA (probable to be active) value predicted by the Way2Drug PASS server. Pa value (probability to be active) is a value that describes the potential of a compound being tested; this value is obtained by comparing the data structure inputted with the existing database (13). If the Pa value is more than 0.7, this indicates that the compound is predicted computationally to have a high potential and have had successful laboratory tests. Whereas if the Pa value is more than 0.3 but less than 0.7 then the compound can be computationally assessed for its activity, but laboratory tests have not been proven or have little potential. If it is less than 0.3, the mixture is computationally challenging to assess, and laboratory tests have low potential. Based on PA analysis, surfactine has considerable potential as a glycopeptide-like antibiotic, with a value of 0.831 (Table 3).

**Table 3.** The Pa value (Probability to be active) of surfactine

**Activity**	**Score**
Antibiotic Glycopeptide-like	0.831
Cell adhesion molecule inhibitor	0.461
Saccharopepsin inhibitor	0.477
Antibacterial	0.501
Antifungal	0.735
Sepsis treatment	0.63

The result of computational docking supported the in vitro results. The *in vitro* results showed that the antimicrobial activity of *B. subtilis* strain PATA-5 was higher against gram-negative bacteria *(E. coli)* when compared to Gram-positive bacteria *(S. aureus)*. The result of computational docking showed that *B. subtilis* contains surfactin that caused leakage and lysis of bacterial lipid membranes, so Gram-negative bacteria such as *E. coli* are more sensitive to surfactin compared to Gram-positive bacteria *S. aureus.*

## DISCUSSION

Testing of *B. subtilis* strains PATA-5 isolates against pathogenic bacteria was done by testing the inhibitor activity of its culture media in the exponential and stationary phases. Some reports have indicated that the inhibitory activity of lactic acid bacteria occurs in the initial exponential phase and continues into the stationary phase (12). The time chosen to represent the two stages was hour 7 post-inoculation for the exponential phase and hour 21 for the stationary phase. As mentioned in our results in Fig. 1, Xie et al. (9) suggested that an inhibition zone with a firm periphery indicates whether the isolate can produce metabolites that are bactericidal. Conversely, inhibition zones with blurred (not firm) edges are categorized as bacteriostatic, i.e., antibacterial components that are only able to inhibit the growth of bacterial test cells (without killing them), so that some test bacteria remain alive in the clear zone. The character of the bacteriostatic LAB-compound inhibition zone has been shown in the study of Xie et al. (9) who found LAB isolates from the Muscovy duck digestive system were able to significantly inhibit *S.* Thypi*, S. aureus, E. coli* and *B. cereus*. Elayaraja et al. (14) found significant bactericidal properties of *L. delbreukii* against *E. coli, S.* Thypi, *S. aureus, B. subtilis* and *K. pneumoniae.*

The potential of *B. subtilis* strain PATA-5 as a natural preservative and biocontrol against pathogenic bacteria was assessed in comparison with tetracycline. The antibiotic tetracycline is a very common food additive in livestock; one study found that it was used 43.15% of the time in food additives and growth promoters for cattle, when compared with aminoglycosides (27.31%), β-lactams (21%) and macrolides (8.42%) (15). Low prices and easy availability are the main reasons why tetracycline is an increasingly popular promoter of growth and treatment and prevention of livestock diseases on a large scale (11).

Elayaraja et al. (14) have categorized tetracycline as an antibiotic that has a broad spectrum of activity against various Gram-negative and Gram-positive bacteria. Meanwhile, differences in LAB-compound inhibition zones against pathogenic bacteria have been reported in the study of Nhung et al. (16), who found that LAB was able to produce extracellular compounds that are antimicrobial and have a greater inhibition zone against Gram-negative bacteria (*Salmonella* serotype Newport), when compared with Gram-positive bacteria *(Listeria monocytogenes)*. Likewise, Heerklotz and Seelig (15) reported that bacteriocin-producing isolates of LAB isolated from Cairo and Giza, Egypt were more effective against Gram-negative pathogenic bacteria (*E. coli* 0157: H7, *S.* Typhimurium, and *P. aeruginosa*) compared to Gram-positive (*S. aureus, L. monocytogenes* V7 and *B. cereus*). Olowe et al. (17) reported the same result, finding that LABs isolated from Indian pickles had a higher antimicrobial activity against Gram-negative bacteria, namely *E. coli* and *S.* Typhimurium, ranging from 16–40 mm and 30–45 mm, respectively, when compared to Gram-positive bacteria from *Bacillus cereus*, ranging from 12–34 mm. The effectiveness of LAB against Gram-negative and Gram-positive bacteria is also caused by the capacity of LAB to produce bacteriocin, and other mechanisms that are specific to the antimicrobial compounds LAB against the cell wall of the test bacteria.

The diameter of the inhibitory zone presented in Table 2 shows that the maximum inhibition of *B. subtilis* strain PATA-5 against *E. coli* and *S. aureus* is at stationary phase, at 21 hours incubation. In contrast to Ripert et al. (4), who reported *B. subtilis* was capable of producing antimicrobial compounds at a higher level (maximum) at the end of the exponential phase. This difference is related to the activity of LAB in producing different antimicrobial main metabolites in each phase of its growth. Sharma et al. (18) and Husain et al. (19) reported maximum lactic acid production by LAB during the stationary phase. Meanwhile, other antimicrobial components such as bacteriocin are produced at the beginning of the stationary phase (2, 12, 13) reported that bacteriocin has a broad spectrum as a maximally produced antimicrobial compound during the stationary fermentation phase.

Intensive and diverse use makes tetracycline residues easily enter the human body via their use in treatment or prevention of livestock diseases and food products. About 27% of broilers thighs from Vietnam were found to contain tetracycline residues (16). The presence of tetracycline (e.g., oxytetracycline, chlortetracycline, and tetracycline) and other antibiotic residues such as aminoglycosides and fluoroquinolone (envoplakin and ciprofloxacin) were also found in chicken meat in New Delhi (18). The presence of tetracycline residues can trigger negative impacts on human health such as gastrointestinal dysfunction, renal insufficiency, and mucosal tissue deformation (20). Various allergies and other adverse effects, due to their physiochemical properties and toxicity are also caused by tetracycline residues through oxidation, reduction, hydrolysis and conjugation (21).

The ability of strains of lactic acid bacteria, as probiotic agents, to inhibit pathogenic bacteria, such as *E. coli, S. aureus, P. aeruginosa, S.* Typhi, *B. cereus, P. mirabilis* and *K. pneumoniae* (22); *E. coli*, *Pseudomonas* sp., *S.* Typhi, *S.* para-Typhimurium B, *Clostridium* sp., *S. aureus, Streptococcus* sp., *B. megaterium* ID 07817, *B. megaterium* ID 07818 and *L. ivanovii* (14); *E. coli, P. aeruginosa, S. typhi, S. aureus* and *B. cereus* (6), has been widely reported. These results are in line with this study, which found *B. subtilis* strain PATA-5 could inhibit *E. coli* and *S. aureus.*

The difference in the character of *B. subtilis* strain PATA-5 against pathogenic bacteria is closely related to the mechanism of inhibition and production of metabolic compounds that are antimicrobial. Castellano et al. (21) suggested that the antagonistic properties of LABs are carried out by reducing intracellular pH and accumulating organic acids into pathogenic bacterial cells to dissociate into the cytoplasm. The same results were reported by Markowiak and Śliżewska (8), regarding the potential of LAB as a probiotic that can inhibit the growth of pathogenic bacteria by reducing the pH of the environment, competitiveness between cells, and the production of specific protein complex compounds (bacteriocin).

Mokoena (3) has reported bacteriocin production by LAB as peptide compounds that have bacteriostatic or bactericidal activity against related or unrelated organisms. The results of the study of Markowiak and Śliżewska (8) found *Pediococcus acidilacticci* and *Streptococcus thermophilus* had bacteriostatic properties against *S. aureus* ATCC 25932 and bactericidal properties against *P. acidilacticci* BL20. Also, Jones et al. (2) reported that inhibition of LAB against pathogenic bacteria is closely related to the accumulation of significant metabolites such as organic acids (lactic acid and acetic acid), ethanol and carbon dioxide. Some low molecular weight metabolites produced by LABs are antimicrobial substances such as fatty acids, re-uterine, re-utericycline, diacetyl, formic, benzoate, acetoin, H_2_O_2_, antifungal compounds such as propionate, phenyl-lactate, hydroxyphenyl-lactate, and fatty acids 3-hydroxy (21).

As with the antibiotic compounds produced by LABs, the mechanism of tetracycline in inhibiting pathogenic bacteria is via binding to the 30S ribosome subunit. It happens through the interaction with 16S rRNA and preventing the installation of amino-acylated tRNA or stopping the assembly of polypeptides, so that tetracycline exposure can reduce the rate of cell division in the target (22). However, the interactions that occur between the two can also trigger the resistance of pathogenic bacteria to antibiotics, as shown in Sharma et al. (18), who found *E. coli* and *S. aureus* populations with resistance to tetracycline compounds.

Several mechanisms of resistance possessed by pathogenic bacteria to antibiotic compounds are efflux, ribosomal protection, and enzymatic inactivation (Park et al., 2017). Heerklotz and Seelig (15) have identified the mechanism of resistance of *S. aureus* to many antibiotic compounds that work by activating the efflux proteins tetA (K) and tetA (L) and ribosome protection through tetA (M). Identification of tetracycline antibiotic resistance expression genes against *E. coli* was also carried out by Olowe et al. (17), who found that tetA and tetB as genes that have high prevalence and the primary determinant of *E. coli* resistance to tetracycline antibiotics. The results of the study by Moller et al. (23) (2016) also found *E. coli* resistance to tetracycline, which was expressed by tetR and tetA genes.

*Bacillus substilis* can produce natural cyclic lipopeptide antibiotics, namely surfactin and plipastatin. Based on research conducted by Heerklotz and Seelig (15), surfactin can inhibit the growth of *S. aureus*, and reduce PSM (phenol-soluble modulin) levels. Phenol-soluble modulins (PSM) are protein toxins that can dissolve in phenols and are produced by staphylococci. Surfactin acts to inhibit the growth and development of other organisms. The microbial function of surfactin is as an antiviral, antibiotic, signaling paracrine in the development of biofilm function. Surfactin is predicted to interfere with the integrity of the membrane.

## CONCLUSION

The identification results using the 16SDNA method showed that the bacteria that were isolated from Takalar Regency, South Sulawesi, Indonesia were *B. subtilis* strain PATA-5. The response of inhibition of antimicrobial compounds produced by *B. subtilis* strain PATA-5 maximum in the stationary phase. The bactericidal properties of *B. subtilis* strain PATA-5 were categorized as strong against Gram-negative *E. coli. B. subtilis* strain PATA-5 is capable of producing natural antibiotic cyclic lipopeptides, namely surfactin, which has the potential to replace synthetic tetracycline antibiotics.
